# Application of Gene Editing Technology in Resistance Breeding of Livestock

**DOI:** 10.3390/life12071070

**Published:** 2022-07-18

**Authors:** Sutian Wang, Zixiao Qu, Qiuyan Huang, Jianfeng Zhang, Sen Lin, Yecheng Yang, Fanming Meng, Jianhao Li, Kunli Zhang

**Affiliations:** 1 State Key Laboratory of Livestock and Poultry Breeding, Guangdong Key Laboratory of Animal Breeding and Nutrition, Institute of Animal Science, Guangdong Academy of Agricultural Sciences, Guangzhou 510640, China; wstlyt@126.com (S.W.); quzixiao123@163.com (Z.Q.); huangqqiu2022@163.com (Q.H.); fdyangyecheng@163.com (Y.Y.); mengfanming@gdaas.cn (F.M.); jianhao63@sina.com (J.L.); 2 Institute of Animal Health, Guangdong Academy of Agricultural Sciences, Guangdong Provincial Key Laboratory of Livestock Disease Prevention Guangdong Province, Guangzhou 510640, China; zhang-jianfeng@139.com; 3 Sericultural & Agri-Food Research Institute, Guangdong Academy of Agricultural Sciences, Guangzhou 510610, China; linsen@gdaas.cn; 4 Maoming Branch, Guangdong Laboratory for Lingnan Modern Agriculture, Maoming 525100, China

**Keywords:** genome engineering, livestock breeding, pigs, cattle, sheep

## Abstract

As a new genetic engineering technology, gene editing can precisely modify the specific gene sequence of the organism’s genome. In the last 10 years, with the rapid development of gene editing technology, zinc-finger nucleases (ZFNs), transcription activator-like endonucleases (TALENs), and CRISPR/Cas9 systems have been applied to modify endogenous genes in organisms accurately. Now, gene editing technology has been used in mice, zebrafish, pigs, cattle, goats, sheep, rabbits, monkeys, and other species. Breeding for disease-resistance in agricultural animals tends to be a difficult task for traditional breeding, but gene editing technology has made this easier. In this work, we overview the development and application of gene editing technology in the resistance breeding of livestock. Also, we further discuss the prospects and outlooks of gene editing technology in disease-resistance breeding.

## 1. Introduction

Livestock production plays an essential role in current social life. Livestock products, such as meat, eggs, milk, and fur, are necessities of human life. Foods from these livestock provide a large quantity of energy and high-quality protein [1]. Therefore, an efficient, healthy, and safe livestock farming industry is closely related to global food safety and public health security. The global epidemics of avian flu, African swine fever, and other animal diseases have made us realize that these infectious livestock diseases badly affect animal health welfare, livestock production, social economy, and human health safety. For a long time, vaccination and antibiotic feeding were the main strategies for disease control in livestock production. However, long-term use of antibiotics causes environmental pollution and increases resistance to harmful microorganisms and aggravated infections [2]. Moreover, there is a lack of effective vaccines for some infectious diseases [3]. The occurrence of diseases is not only related to livestock feeding and production environment but is also affected by genetic factors. Therefore, when traditional methods prove ineffective, many researchers have turned to improving animal genomes to enhance their disease-resistance.

The disease-resistance of livestock has a direct bearing on their fitness, which significantly affects growth and reproduction. The interaction between pathogenic microorganisms and the host immune system affects disease infection and transmission. Many diseases are related to genetic factors [4,5]. The host’s innate immunity is the first line of defense against pathogen infection, and enhancing innate immunity can offer non-specific protection against widespread infection of pathogenic microorganisms [6]. It is also demonstrated that many innate immune traits are genetically controlled in livestock [7]. Therefore, innate immune-related genes are the primary goals of disease-resistant breeding. On the other hand, more and more antivirus and host-receptor proteins have influenced infection by specific pathogens [8,9,10]. Controlling the level of these proteins can also achieve resistance to particular pathogen infection.

**Figure 1:**
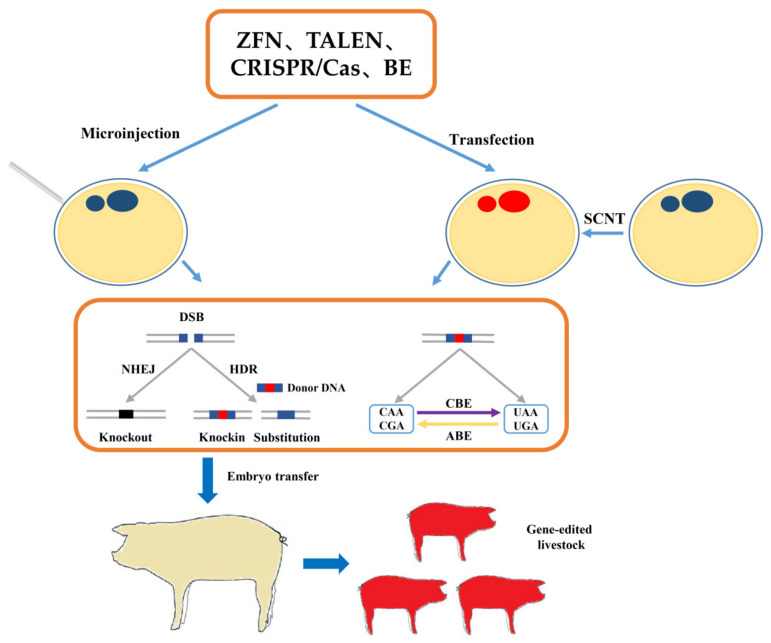
Schematic diagram of gene editing in resistance breeding of livestock. ABE, adenine base editors; BE, base editing; CBE, cytosine base editors; DSB, double-stranded break; HDR, homologous directed repair; NHEJ, non-homologous end junctions; SCNT, somatic cell nuclear transfer; TALEN, transcription activator-like endonucleases; ZFN, zinc-finger nucleases.

The disease-resistance traits have been considered the target traits for the genetic improvement of livestock. Using modern genetic methods and molecular biology techniques to improve livestock disease-resistance and immune capacity could improve livestock production efficiency and promote the healthy development of the livestock industry. Traditional breeding methods usually have shortcomings, such as long cycles, low efficiency, and prediction difficulty [11]. Firstly, a large population is needed to obtain a superior trait, and meanwhile, continuous selection and assortative mating are needed to stabilize this trait. Many of today’s superior agricultural animals have gone through decades of breeding (such as Landrace swine and Holstein cows). More than that, it is hard to achieve rapid improvement when some important economic properties have reached a certain level [12]. We should admit that traditional breeding has entered a bottleneck, especially in a rapidly growing population with limited resources. More importantly, the traits selected by traditional breeding methods must have existed in the breeds. Livestock genetic resources have severely limited this process. Additionally, because disease-resistance traits are hard to observe and there is a substantial potential risk of persistent infection, it is difficult for conventional breeding methods to work effectively. Therefore, gene editing technology to promote disease-resistance breeding of livestock has become a current research hotspot. In recent years, zinc-finger nuclease (ZFN), transcription activator-like effector nuclease (TALEN), Clustered regulatory interval palindromic repeats/CRISPR-related (CRISPR/Cas), and base editing (BE) technologies are advancing rapidly. The most notable features of these technologies are their high efficiency and precision. By using gene editing technology, targeted mutations can be made in specific genes, and novel lines of animals with valuable phenotypes can be generated (Figure 1). Meanwhile, gene editing technology can effectively avoid the gene chain effect and reproductive isolation between species. Gene editing effectively solves time-consuming, high-cost, and low-efficiency problems. Scientists have generated livestock for disease-resistant purposes, including PRRSV-resistant swine, Mycobacterium-resistant cows, ALV-resistant chickens, etc. However, there are very few known effective host disease-resistance genes (mainly regarding CD163, RELA). The main challenge is the discovery of gene editing disease-resistance targets, which can also be achieved by gene editing technology itself. Here, we reviewed the research progress of gene editing techniques in disease-resistance breeding of livestock and prospected its development influence on breeding to provide a reference for future research.

## 2. Gene Editing Tools and Their Application in Farm Animals

### 2.1. Zinc Finger Nuclease

Zinc finger nucleases consist of tandem zinc finger-binding motifs and restriction endonuclease FokI [13]. Zinc finger proteins (ZFPs) are responsible for identifying and binding specific DNA sequences, and Fokl performs the cutting function. Zinc finger protein was first discovered in 1983 in transcription factor IIIA, a transcription factor in Xenopus laevis oocytes [14]. A zinc finger unit consists of about 30 amino acids of conserved ββα configuration, of which the Cys2-His2 zinc finger domain is one of the most common DNA-binding motif types in eukaryotes [15,16]. Since one ZFP unit recognizes only three DNA bases, it usually takes more than three zinc finger domains to identify a specific DNA sequence. The design of DNA-binding proteins depends on the modular structure of zinc finger proteins [17]. The DNA binding domain of ZFPs has a unique surface structure complementary to the DNA double helix. This particular finger-shaped spatial structure helps to protrude into the large groove of the DNA double helix, making it possible to contact specific DNA bases [18]. The following conditions must be met for the binding of zinc finger and DNA: (1) the α helix of the zinc finger protein is located in the large groove; (2) the area where the zinc finger protein carries the positive charge is close to the phosphoric acid skeleton; and (3) the joint structure between the zinc finger is relatively fixed. The FokI then breaks the nucleotide chain downstream of the binding site. When the target regions of two ZFNs are 6–8 bp apart, FokI of ZFNs can induce double-stranded breaks (DSBs), which further cause DNA repair processes, including error-prone non-homologous end junctions (NHEJ) and comparatively accurate homologous directed repair (HDR) [19].

In the past decades, ZFN technology has been used in genetic analysis, animal improvement, and animal model preparation [20,21]. In 1996, Kim et al., designed the first ZFNs with in vitro activity [22]. Until 2009, the Medical College of Wisconsin, Sangamo Biosciences, Sigma-Aldrich, and other institutions successfully used ZFN technology to create the first targeted gene knockout rat, which could produce offspring with the same genetic mutation [23]. In 2010, a Japanese group used ZFNs to successfully knock out the exogenous EGFP gene in porcine somatic cells, demonstrating that the ZFN-KO system could be applied to domestic animals [24]. ZFNs have shown significant breakthrough and potential compared with traditional transgenic technology. ZFN has high specificity and improves the efficiency of genetic modification by tens of thousands of times. However, as the first generation of gene editing technology, it still has some inevitable shortcomings. First of all, its gene editing efficiency is still low. The gene targeting rate of ZFNs in porcine cells hardly exceeds 5% [25]. Secondly, the design and assembly of the ZFN is a challenge for many researchers, and the effective commercial ZFN reagents are still very expensive. Additionally, because the specificity of adjacent ZF motifs is not independent of each other, the targeting activity of ZFN technology remains unpredictable [26,27]. These shortcomings limit the popularization and development of this technology. Currently, the research on zinc finger proteins mainly focuses on further identifying zinc finger proteins related to gene expression and regulation and analyzing their structure to establish models of their interaction with DNA.

Whyte et al., generated the first ZFN-mediated knockout pig of an enhanced green fluorescent protein eGFP transgene [28]. After transfecting the ZFN plasmids into porcine fibroblasts, eGFP knockout cells (~0.1% of sorted cells) were selected by fluorescence-activated cell sorting. Subsequently, these ZFN-mediated mutant cells were used as donors for somatic cell nuclear transfer and embryo transfer. The research generated several pigs with KO eGFP fluorescence. ZFN-mediated myostatin (MSTN)-KO pigs showed increased muscle mass, decreased fat, and muscle hypertrophy phenotype [20]. ZFN-mediated endogenous peroxisome proliferator-activated receptor-γ KO-pigs were new animal models for studying cardiovascular disease [21]. Generating α-1,3-galactosyl-transferase gene biallelic KO pigs by ZFN made xenotransplantation possible [29]. Although the current KO pigs are not suitable for significant long-term xenotransplantation, we considered that this is the first step toward xenotransplantation using pig organs. Liu et al., successfully integrated the lysostaphin coding vector into the endogenous β-casein locus by ZFNs in bovine fetal fibroblasts. These gene-edited cows could secrete milk containing lysostaphin, which helped relieve mastitis [30]. Due to different laws and regulations, these gene-edited animals are only commercially available in a few regions.

### 2.2. Transcription Activator-like Effector Nucleases

TALENs have a similar structure to ZFNs. They also consist of a DNA-binding domain and cleavage domain [20]. Moreover, a pair of TALENs are required to induce DSBs [31]. The DNA-binding domain of TALENs is named transcription activator-like effector (TALE), which was first discovered in effector proteins secreted by Xanthomonas [32]. The DNA-binding domain includes 30 tandem repeats of 33–35 amino acids, with each repeat domain identifying individual base pairs [33]. Theoretically, TALE could target any DNA sequence [34]. Researchers linked an artificial TALE with the single-strand DNA-cleaving domain of FokI to form a powerful tool called TALEN that combines the characteristics of TALE and FokI. Compared to ZFNs, TALEN technology is simple to operate and low cost. The repeat variable diresidues (RVDs) NN, NI, NG, and HD identify G, A, T, and C [34]. Like ZFNs, TALENs can also edit specific DNA sites by producing DSB-mediated NHEJ and HDR. However, since the structure of TALEN is relatively simple and its recognition of DNA sequence is more precise, TALEN shows increased gene editing efficiency and decreased toxicity, cost, and off-target risk. Numerous studies have shown that TALEN pairs could efficiently trigger KO of target genes in many livestock, with a 20–60% knockout efficiency [35,36]. Surprisingly, TALEN-mediated gene knock-in efficiency exceeds 30% at some loci [36,37,38].

Although using ZFN to modify livestock genomes has proven to be an effective strategy, this technique is limited by its complex design, high cost, and available targets [39]. Many research groups quickly applied TALEN technology to livestock genome modification and genetic improvement based on the advantages of TALEN technology. Carlson et al., successfully used TALEN to modify livestock genomes, proving its excellent application potential [35]. The study showed that 64% TALENs had high activity in primary porcine cells. Injecting TALEN mRNA directly into livestock zygotes resulted in the knockout of target genes in 75% of embryos (with 29% in swine and 43–75% in bovines). It successfully produces MSTN-KO swine, bovine, and lamb using TALENs [40,41,42]. The TALEN’s efficiency was no less than 10% among the livestock, and there were significant changes in muscle phenotypes. Moreover, TALEN has been successfully used to generate pig models of cardiovascular diseases with biallelic modifications of the *LDLR* gene [35]. Interestingly, Cui et al., explored knockout β-Lactoglobulin gene followed by human lactoferrin gene knock-in using TALENs in goats [43]. This research showed that gene editing via TALEN-mediated HDR could generate genetically engineered livestock, also used as mammary gland bioreactors to produce specific products efficiently.

### 2.3. Clustered Regularly Interspaced Short Palindromic Repeats/CRISPR-Associated Protein

Compared with the gene editing technology described above, CRISPR-Cas9 may be a more powerful tool for livestock genetic improvement. Among archaea and bacteria, there are clustered regularly interspaced short palindromic repeats (CRISPRs) that act as an acquired immune system defense against invading DNA contaminants through DNA or RNA interference [44,45]. In 1987, Japanese scientists identified this unusual structure in the genome of *E. coli*, containing a series of repeated fragments separated by a unique sequence of intervals [46]. Until 2007, experiments on *Staphylococcus thermophilus* revealed that this immune system is coordinated by CRISPR and Cas proteins [47]. The most commonly used CRISPR/Cas9 gene editing system is an artificially modified type II CRISPR system. This system consists of crRNA, trans-activating crRNA (tracrRNA), and nuclease Cas9. The crRNA/tracrRNA complex guides nuclease Cas9 to trigger DSB at the crRNA-paired DNA sequence target site. The CRISPR/Cas9 system can target any 20-nucleotide that lies immediately 5′ of an NGG protospacer adjacent motif (PAM) sequence. Compared to ZFNs, TALENs, and CRISPR/Cas systems are more straightforward, economical, and efficient [48]. However, the potential risks of off-target and genetic variation are brought into focus. 

The CRISPR/Cas9 system is so simple to design and use that almost any biology lab can use it to conduct research. By designing and synthesizing a short nucleic acid base on the sequence near PAM of the target gene and attaching it to the corresponding vector (such as PX330, PX459) by enzyme digestion, researchers can use the CRISPR/Cas9 system to modify specific genes. This technique has been widely studied and applied to improve livestock heredity, reproduction, and nutrition levels [49]. CRISPR/Cas9 was used to improve the meat production trait of various livestock by efficiently knocking out MSTN [50,51,52]. CRISPR/Cas9-mediated knockout of the stearoyl-CoA desaturase one gene can improve the nutritional value of milk [53,54]. CRISPR/Cas9-mediated knockout of fibroblast growth factor 5 gene improves growth and hair traits in goats and sheep [55,56]. CRISPR/Cas9-mediated CD163/pAPN knockout pigs or NRAMP1 knock-in cattle show more excellent disease-resistance [8,57,58]. Moreover, numerous animal models constructed with the CRISPR/Cas9 system serve as the mammary gland bioreactors to produce large quantities of valuable human protein products [59,60,61]. Additionally, many genetically modified pigs established by using CRISPR/Cas9 are suitable donors for disease models and xenotransplantation [62,63,64,65]. These studies show that CRISPR/Cas9 has great potential to improve modern livestock breeding systems.

### 2.4. Base Editing Systems

Single nucleotide variants (SNVs) are an important genetic basis for trait variation in livestock and the leading cause of 2/3 of human diseases [66]. Therefore, developing an accurate and efficient tool for single-base substitutions is very important and urgent. Researchers developed the base editing system in this context, a new target gene editing technology developed based on the CRISPR/Cas system. Unlike CRISPR/Cas, base-editing systems use nucleotide deaminases and artificially modified Cas proteins to replace bases at the target site by creating a single incision in the double-strand. According to different base modification enzymes, the base editing systems consist of cytosine base editors (CBE) and adenine base editors (ABE), which can achieve C–G to T–A, and A–T to G–C substitutions, respectively [67]. The emergence of BE system provides a powerful tool for precise genome editing, which has many advantages. Firstly, BE-mediated gene editing is independent of DSBs. DSBs-triggered NHEJ repair pathway is unpredictable and may induce unessential indels production. In addition, excessive DSBs will result in cytotoxicity [68]. On the other hand, researchers also do not need to screen for highly active sgRNA and Cas nucleases. Secondly, the BE system does not require donor DNA, an indispensable component of the HDR repair pathway. Designing efficient donor DNA for livestock and delivering donor DNA efficiently into livestock cells are practical challenges [69].

Based on these advantages, some teams have already used the BE systems to carry out genetic improvement work in livestock. Xie et al., first used the CBE system (BE3 and hA3A-BE3) to trigger single base substitutions efficiently at multiple sites in pig cellular, embryonic, and individual levels simultaneously. The CBE system was first used to generate base editing pigs to establish larger animal models [70]. Subsequently, many groups have used CBE and ABE to knock out different porcine genes, such as GGTA, MSTN, CD163, GHR, and IGF2, for pig trait improvement [71,72,73,74,75]. BE3 and ABEmax generated goats and sheep with Socs2, GFG5, and BMPRIB mutations in Northwest A&F University [76,77,78]. Unlike DSB-mediated mutation, BE-mediated mutation is closer to precise control. These results also suggest that the BE systems can efficiently improve livestock production, reproductive, milk-producing, and wool-producing traits. In conclusion, compared with previous gene editing techniques, a base editing system can significantly improve efficiency and accuracy in livestock breeding, which is expected to boost genetic improvement for large animals.

## 3. Application of Gene Editing Technology in Disease-Resistance Breeding of Livestock

Traditionally, the main methods of livestock breeding include crossbreeding and selective breeding. Almost all the livestock we raise today were bred through crossbreeding and long-term selection [79]. It is indisputable that the production performance of these breeds bred by traditional means has been significantly improved compared with the original breeds. However, traditional breeding methods have been unable to introduce or improve high-quality genes quickly and accurately without introducing bad genes (meaning the genes which would result in trait deficiencies in livestock production such as infertility, susceptibility, low growth, etc.) [80]. Furthermore, in conventional breeding methods, it is hard to select complex traits that are difficult to observe and measure, such as disease-resistance traits. Moreover, measuring after the challenge experiment is costly and adversely affects livestock production and welfare. Meanwhile, the high disease-resistance pursued by large-scale livestock breeding antagonizes some production traits. Additionally, the resistance traits of many diseases are controlled by multiple genes, and the breeding improvement cycle is so long. Therefore, the development of resistant livestock breeds has been slow [81]. At the same time, breeding with genome editing can quickly cultivate new livestock varieties with disease-resistance traits by directly deleting disease susceptibility genes and pathogens receptor genes or inserting disease-resistance genes. We can also give livestock genetic traits for disease-resistance that are not available from natural genetic resources, resulting in new livestock varieties that are not available through traditional breeding.

Swine are one of the most important livestock resources and are considered the most suitable animal model for biomedical research and xenotransplantation because they share similar physiological and genetic characteristics with human beings [62,82]. Therefore, it is crucial to maintain the healthy and stable development of the swine industry. At present, the swine industry is threatened by a variety of viruses, including ASFV (African Swine Fever virus), PRRSV (Porcine Reproductive and Respiratory syndrome virus), PEDV (porcine epidemic diarrhea virus), TGEV (infectious gastroenteritis virus), CSFV (classical swine fever virus), PRV (pseudorabies virus), etc. [83]. African swine fever (ASF) first broke out in the 1950s in Europe and Latin America. A report indicated that the outbreak of ASF resulted in tens of billions of dollars in losses in the U.S [84]. In 2018, ASF was introduced to China, the world’s largest pig-raising country. Since ASF is highly lethal and contagious, its infection can devastate the local pig Industry [85]. The impact on China’s economy would be many times greater than that of the U.S. Since there is still a lack of effective vaccines and drugs against the disease, many researchers are currently trying to improve pig resistance by modifying the host’s genome [86]. Since ASFV infects and replicates in porcine alveolar macrophages (PAMs), many disease-resistance studies have focused on identifying and knocking out macrophage membrane receptors. Initially, ASFV only infects CD163^+^ monocytes but not CD163^−^ monocytes, suggesting that CD163 may be a key membrane receptor during ASFV infection [87]. In 2017, Popescu et al., generated CRISPR/Cas9-mediated CD163-knockout pigs [88]. It is a pity that this genetically edited pig is still ineffective against ASFV infection. The researchers suggest that there may be other receptors on the membrane of porcine macrophages that can recognize ASFV except for CD163. By comparing the gene sequences of domestic pigs and warthogs, Palgrave et al., found that the polymorphic variation of RELA may affect pig resistance to ASFV [89]. A British team generated RELA-modified domestic pigs with the warthog RELA orthologue [90]. Although the gene-edited pigs did not show resistance to ASFV, they have a lower viral load in blood and nasal secretion [91].

PRRS causes severe reproductive dysfunction in sows and transplacental transfer of PRRSV to the fetus in the third trimester of pregnancy, resulting in miscarriage, early delivery, increased stillbirth numbers, and neonatal weakness [92]. Boars infected with PRRSV develop anorexia, lethargy, and reduced sperm quality [92,93]. In 2007, Calvert established several stable cell lines expressing CD163, and these modified cells were susceptible to PRRSV [94]. The study showed the C-terminal transmembrane anchor domain of CD163 is a critical structure that mediates PRRSV infection. Further research found that the expression of CD163 in PAM affects the replication efficiency and subsequent pathogenicity of PRRSV [95]. Remarkably, Whitworth et al., first used CRISPR/CAS9 to generate a CD163-knockout pig and found that the genome-edited pig is fully resistant to PRRSV infection [96]. After a challenge with PRRSV, these pigs showed no obvious PRRSV-related clinical symptoms, and neither PRRSV nor its antibodies were detected in pig serum. This is the first time that gene-edited breeding for livestock disease-resistance has solved a problem that neither vaccines nor selection programs can solve perfectly. Since then, several teams have generated a variety of CD163-knockout pigs using gene editing techniques. These CD163 null mutations are completely resistant to various PRRSV isolates [8,97,98,99].

CSFV, which belongs to the plague virus genus of Flaviviridae, is a highly contagious porcine disease that endangers the swine industry [100]. Although some vaccines are effective against CSFV infection, some researchers still hope to improve pigs’ resistance to the virus. Zhao et al., used nuclear transfer to generate transgenic pigs expressing human MxA. The results showed that transgenic pigs with overexpression of MxA could inhibit CSFV early point post-infection [101]. In 2019, Xie et al., realized site-specific insertion of the porcine RSAD2 gene (pRSAD2) at the porcine ROSA26 (pROSA26) site using CRISPR/Cas9 technology. The pRSAD2-knock-in pig showed resistance to CSFV and PRV infection [102]. Moreover, a Chinese research group has inserted shRNAs into the pRosa26 locus and generated transgenic pigs by CRISPR/Cas9-mediated knock-in strategies, which are resistant to CSFV infection [103,104].

Porcine epidemic coronaviruses (PECs) mainly infect the intestines of newborn piglets and cause acute diarrhea, vomiting, dehydration, and high mortality [105]. Porcine aminopeptidase-N (pAPN), a key receptor of various PECs, may be a potential breeding target to improve porcine PECs-resistance [106,107,108]. CRISPR/Cas9 and CBE have been used to generate pAPN-null pigs resistant to TGEV and PDCoV, but not PEDV. Moreover, these resistant gene-edited pigs’ growth and reproductive traits were not significantly different from wild-type pigs [8,72,109,110].

Cattle are an essential source of high-quality meat and dairy products, so their industry needs to be kept healthy and efficient. Mastitis is the most severe disease in dairy cows and causes considerable losses to the dairy industry. Preventing and treating mastitis has been a problem in the dairy industry. Liu et al., inserted the lysostaphin gene and human lysozyme gene into the β-casein locus of the cow genome using ZFNs. They then generated transgenic cows by somatic cell nuclear transfer. The gene-edited cows could secrete lysostaphin or human lysozyme, which could kill *Staphylococcus aureus* in milk [30,111]. In 2015, the mouse SP110 gene was inserted into MAT1A and SFTPA1 locus of Holstein-Friesian dairy cows genome using TALEN [10]. The transgenic cattle showed increased resistance to M. bovis infection. Subsequently, this research group integrated the NRAMP1 gene into the FSCN1-ACTB (F-A) locus and the bovine homology of the mouse Rosa26 locus using the CRISPR/Cas9 system, respectively. These NRAMP1 transgenic cows showed increased resistance to tuberculosis [58,112]. ZFNs have been used to produce a cattle homozygous for the Q(−5)G substitution, which expressed CD18 without the signal peptide. This precise-engineered ruminant was shown resistant to *M. haemolytica*-caused pneumonia [113]. 

Research on modifying sheep genomes using gene editing techniques has improved growth, hair, and milk-producing traits. A recent report suggested that used CRISPR/Cas9 system to modify HYAL2 and PrP genes was expected to generate disease-resistant lambs [114]. 

Avian leukaemia virus (ALV) infection is believed to be responsible for serious commercial losses in the poultry industry. It has been confirmed that knocking out the ALV receptor gene was expected to confer ALV resistance [115]. In 2020, Koslova et al., successfully generated chNHE1-KO homozygous mutant chicken by primordial germ cell and CRISPR/Cas9 technologies [116]. This transgenic chicken has the ability to fight ALV infection. Chicken avian influenza virus (AIV) is another serious disease that spreads quickly and has high mortality in chickens. CRISPR/Cas9 was used to modify the identified residues within the chANP32A gene to decrease AIV replication in chicken cells [117]. These studies provide a feasible strategy for improving the disease-resistance of livestock and improving their health and welfare (Table 1).

## 4. Prospect of Gene Editing Technology in Disease-Resistance Breeding of Livestock

There is no doubt that the emergence of gene editing technology has produced inestimable value for the progress of agricultural breeding. The new varieties can be generated by DNA base-level editing without limitation to individuals or species. Gene editing technology can break down the limitations of reproductive isolation and enable the host to acquire disease-resistance traits of other species, which cannot be achieved in traditional breeding efforts. The introduction of foreign genes, however, raises new safety and ethical issues that need to be more rigorously argued. The new varieties can achieve the precise integration, deletion, and substitution of genes in the genome of any livestock. Moreover, gene editing technology has significant advantages in improving complex traits that are difficult to observe and determine, such as resistance-disease characters. Although breeding with gene editing has many benefits, some problems remain to be solved. As the first generation of gene editing technology, the efficiency and accuracy of ZFN are significantly higher than that of traditional HDR. However, its limited targets, high costs, complicated design, lengthy screening, and relatively low efficiency have restricted its development and promotion. TALEN technology can surpass ZFN due to its simple design, better editing efficiency, and more flexible target selection. However, the large volume of TALEs proteins may be cytotoxic and increase transfection strategies’ requirements, limiting its application in high-throughput gene editing [31]. However, these two gene editing techniques rely on synthesizing specific binding proteins, and their complicated operation makes their application progress slow. The emergence of the CRISPR/Cas system marks another major leap forward in the mammalian genome’s accurate and efficient specific gene modification. CRISPR/Cas system, which uses nucleic acid to identify targets, has obvious advantages over ZFN and TALEN, which use protein to identify targets. Unfortunately, the CRISPR/Cas system is limited by potential off-target rates, cytotoxicity, strict PAM restrictions, and low HDR pathway efficiency [125,126,127]. Whether these problems can be overcome will affect the future of this technology. Currently, some studies have added NHEJ inhibitors to provide efficiency of the HDR pathway, thus improving repair accuracy [128,129]. With the deepening of CRISPR-related studies, many studies have found that the occurrence probability of the NHEJ repair pathway is closely related to the cell cycle [130]. Therefore, appropriate cell cycle regulation can promote HDR-mediated accurate repair [131,132]. In addition, exogenous DNA, chemically modified sgRNA, and some small molecular compounds can also stimulate HDR [133,134,135]. On the other hand, improving the specificity of CRISPR/Cas systems by optimizing Cas protein and sgRNA can enhance the targeting range and precision [136,137,138]. The characteristics of these gene editing systems used in livestock are summarized in Table 2. When genome editing technology is used in human disease therapy, we need to carefully consider its off-targeting, which may pose potential health risks such as oncogenesis. However, we can avoid the off-targeting risk by genome deep sequencing before SCNT and embryo transfer, guaranteeing our gene editing livestock is in line with expectations, particularly the knockout mutation. Significantly, when we introgress foreign genes into livestock genomes, we need to consider the locus of gene integration, the efficiency of gene expression and potential biosafety issues. Studies have shown that the CEP112 locus had higher knock-in efficiency than the ROSA26 locus, suggesting we discover safe and effective knock-in loci [10,58,139]. Therefore, every practitioner should be aware of the importance of early screening. Additionally, constructing multiple sgRNA expression vectors using the gene editing system, which can simultaneously modify multiple genes in the livestock genome, will be the main direction of gene editing technology in the resistance breeding of livestock [8].

Finding and screening out genes that can affect the ability of livestock to resist disease is a difficult task in disease-resistance breeding. Although we have found that some host genes (such as pig CD163 and APN) play key roles in influencing infection, few are known to be effective. How to find effective host disease-resistance/susceptibility genes has posed great challenges to many researchers. Traditional research on disease-resistance breeding relies on disease-resistant populations in nature or the results of basic research on pathogen-host interaction. Therefore, identifying an effective disease-resistance locus in livestock often requires time, energy, and money, and carries a potential risk of disease spread. The establishment of CRISPR/Cas9 genome-wide scanning technology provides a powerful tool for the high-throughput screen of the disease-resistance gene. High throughput and systematic, unbiased, targeted genome-wide editing techniques are significant to fully explore the function and genetic regulation of a disease-resistance gene in livestock. The most important factor in implementing CRISPR-Cas9 library screening is selecting an appropriate screening model, and pathogen infection of host cells usually leads to cell death, which is an ideal screening model. At present, the CRISPR screening libraries mainly include the CRISPR knockout library, CRISPR activation library, and CRISPR inhibition library [140,141,142]. In 2020, a Chinese group developed a porcine genome-scale CRISPR-Cas9 knockout library, which consisted of over 85,000 sgRNA targeting 17,743 protein-coding genes, 11,053 long ncRNAs, and 551 microRNAs [143]. Several previously unreported genes, such as *SLC35B2*, *HS6ST1*, *EMC3*, and *CALR*, required for Japanese encephalitis virus (JEV) infection are highly enriched post-JEV selection. Subsequently, functional verification results showed that these genes were involved in JEV replication. Moreover, the study also identified several metabolic pathways closely related to JEV infection, improving our understanding of JEV infection. Similarly, Sun et al., used a porcine CRISPR knockout library to identify that porcine TMEM41B could serve as a broad-spectrum antiviral target, which played an important role in the early-stage replication of PECs [144]. With the porcine CRISPR knockout library, COG8 and KXD1 genes were identified to play key roles in resistance to influenza virus and PRRSV infection [145,146]. CRISPR/Cas9 genome-wide scanning technology can identify effective disease-resistant mutations that do not exist in nature, which greatly expands the boundaries of disease-resistant breeding. The main bottleneck of this technique is the difficulty of obtaining a suitable screening condition. However, some disease-resistance traits that are unsuitable for cell death and virus replication as screening conditions still lack effective strategies to explore the target genes. Additionally, the repeatability of CRISPR screening libraries for mining functional genes is not stable enough, which may contribute to the quality of the library. The researchers still need to optimize CRISPR screening libraries’ construction, screening conditions, and activity detection in future studies.

The biggest problem with applying and promoting genetically modified agricultural food animals is not the technology itself but government regulations and public acceptance. Currently, only U.S. FDA allows genetically modified livestock to enter the consumer market. The genetically modified products without exogenous genes are exempt from regulation in some countries, including Australia and New Zealand. We have to admit that the development speed of gene editing technology is too fast that most non-professionals do not fully understand and accept it, which has affected the popularization of this technology. However, while PRC and EU countries ban genetically modified agricultural food animals from the consumer market, they still spend a lot of money each year to push the development of this technology. Moreover, it is urgent to reserve relevant technologies and patents in advance in the face of a big potential market and possible food safety. Many practitioners have been sceptical of genetically modified products entering the consumer market over the last decade. Earlier this year, the Chinese government launched a series of policies to push genetically modified crops into the mass market. Predictably, this genetically modified food from livestock may appear on the ordinary dinner table with the gradual perfection of the technology and the continuous improvement in public recognition. We trust that this road is tortuous, but the prospects are bright.

In conclusion, gene editing technology has made great achievements in the disease-resistance breeding of livestock, and this breeding technology will bring sustainable development to the livestock industry. With the improvement of gene editing technology, its safety and superiority will be further guaranteed. Gene editing technology will surely achieve more remarkable results in livestock breeding and provide better and healthier products for all humankind.

## Figures and Tables

**Table 1:** A list of gene-edited livestock for disease-resistant purpose.

Species	Targeted Gene	Disease (Pathogen)	Technique	References
Pig	RELA Substitution	ASF (ASFV)	ZFN	[90,91,118]
Pig	CD163 Knockout	PRRS (PRRSV)	CRISPR-Cas9	[8,96,97,98,99]
Pig	RSAD2 Knockin	CSF, Pseudorabies (CSFV, PRV)	CRISPR-Cas9	[102]
Pig	pAPN Knockout	TGE, Diarrhea of piglets (TGEV, PDCoV)	CRISPR-Cas9	[109]
Pig	CMAH Knockout	Diarrhea of piglets (PEDV)	CRISPR-Cas9	[110]
Pig	Ig-JH Knockout	Pregnancy death (HEV)	CRISPR-Cas9	[119]
Pig	TMPRSS2 Knockout	Influenza (SIVs)	CRISPR-Cas9	[120]
Pig	Antiviral shRNA Knockin	CSF (CSFV)	CRISPR-Cas9	[103]
Pig	PBD-2 Knockin	Pathogens infection (Pleuropneumoniae)	CRISPR-Cas9	[121]
Cattle	Lysostaphin Knockin	Mastitis (*Staphylococcus aureus*)	ZFN	[30]
Cattle	PRNP Knockout	Mad cow disease	CRISPR-Cas9	[122]
Cattle	Human lysozyme Knockin	Mastitis (*Staphylococcus aureus*)	ZFN	[111]
Cattle	Mouse SP110 Knockin	Tuberculosis (*M. bovis*)	TALEN	[10]
Cattle	NRAMP1 Knockin	Tuberculosis (*M. bovis*)	CRISPR-Cas9	[58,112]
Cattle	CD18 Substitution	Pneumonia (*M. haemolytica*)	ZFN	[113]
Goat	FAT-1 Knockin	Cardiovascular diseases	CRISPR-Cas9	[123]
Chicken	NHE1 Knockout	Avian leukosis (ALV)	CRISPR-Cas9	[116,124]
Chicken	ANP32A Knockout	Avian influenza (AIV)	CRISPR-Cas9	[117]

**Table 2:** Features of gene editing tools applicable to livestock genetic improvement.

Tool	Identification of Target DNA	Advantages	Disadvantages
ZFNs	Zinc finger protein	The first generation of gene editing technology	High costs, complicated design, lengthy screening, low efficiency, and limited target
TALENs	TALEs	Flexible target selection, low off-target effects	Complex module assembly, cytotoxicity, difficult to transfect
CRISPR/Cas	sgRNAs	Low costs, easy to design, multiple edits	Potential off-target, PAM limitation, low HDR efficiency
BE	sgRNAs	Independent of DSBs and donor DNA, high efficiency	Potential off-target, unable to achieve base transversions
